# Safety analysis of quinolones use in minors—based on the FAERS database

**DOI:** 10.3389/fmed.2024.1437376

**Published:** 2024-08-29

**Authors:** Yanwei Li, Jing Wang, ChengLiang Wang, Li Chen

**Affiliations:** ^1^Department of Pharmacy, People’s Hospital of Ganzi Tibetan Autonomous Prefecture, Kangding, Sichuan, China; ^2^Department of Pharmacy, Sichuan Mianyang 404 Hospital, Mianyang, Sichuan, China; ^3^Department of Pharmacy, West China Second University Hospital, Sichuan University, Chengdu, Sichuan, China; ^4^Chinese Evidence-Based Medicine Center, West China Hospital, Sichuan University, Chengdu, Sichuan, China; ^5^Department of Pharmacology, Faculty of Medicine and Nursing, University of the Basque Country, Leioa, Spain

**Keywords:** FAERS, minors, quinolones, safety analysis, reporting odds ratio method, proportional imbalance method

## Abstract

**Objective:**

This study utilizes the FDA Adverse Event Reporting System (FAERS) to investigate adverse drug event (ADE) signals linked to quinolones use (ciprofloxacin, moxifloxacin, levofloxacin, ofloxacin) in minors, offering insights for clinical use.

**Methods:**

Minors were categorized into four age groups. ADE reports for these quinolones from the first quarter of 2015 to the third quarter of 2023 were extracted from the FAERS database. Data analysis used reporting odds ratio (ROR) and the MHRA method.

**Results:**

Most ADE cases in minors involved ciprofloxacin (575)and levofloxacin (477). In the infant group, various injury, poisoning, and procedural complication events were more frequently associated with ciprofloxacin, levofloxacin, and moxifloxacin (19.83%, 31.25%, and 100.00%, respectively). In the preschool children group, psychiatric disorders were more frequently reported with levofloxacin and ofloxacin use (59.00% and 47.62%, respectively). Ocular disorders were notably associated with moxifloxacin in the children group (62.50%), In the adolescent group, more gastrointestinal diseases occurred with ciprofloxacin (12.96%).

**Conclusion:**

ADE occurrence with quinolones in minors varies by age. Strict adherence to indications, rational use, avoiding prolonged use, and monitoring for short-term reactions are essential. Enhanced monitoring of interactions and drug education are crucial to reducing ADE.

## 1 Introduction

Quinolones antibiotics, encompassing ciprofloxacin, moxifloxacin, levofloxacin, and ofloxacin, are synthetic antibacterial agents extensively employed in treating infections in adults, including those affecting the urinary tract, intestines, respiratory system, and skin and soft tissues (1). Owing to findings from juvenile animal studies that demonstrate the potential for quinolones to induce cartilage and joint damage (2–4), the utilization of these drugs in pediatric patients is restricted. Most pharmaceutical labels advise against their administration in children and adolescents under 18 years of age (5). Approved indications encompass post-exposure prophylaxis for inhalational anthrax, severe and complicated urinary tract infections, complicated pyelonephritis, plague, and excluding cystic fibrosis, bronchopulmonary infections caused by *Pseudomonas aeruginosa* (6–10). In light of the escalating trend of bacterial resistance, the off-label application of quinolones in pediatric patients is becoming increasingly prevalent in clinical settings. Such off-label applications include the treatment of conditions such as fever with neutropenia, Mycoplasma pneumonia, recurrent and/or resistant acute otitis media, gastrointestinal infections (including typhoid, Salmonella, Shigella infections, and bacterial dysentery), brucellosis, and drug-resistant tuberculosis (11–18). Concurrently, the safety of quinolones administration in this population, particularly with respect to musculoskeletal damage, persists as a significant concern. However, multiple studies have demonstrated that systemic administration of quinolones in children results in a low and reversible incidence of musculoskeletal adverse events (19–23). Other potential ADE, particularly those affecting the nervous and digestive systems, warrant increased attention (9, 24, 25). Most research has predominantly focused on musculoskeletal side effects, with comparatively less attention devoted to other potential systemic ADE associated with quinolones administration. Additionally, there is a paucity of clinical awareness regarding the safety differences associated with the use of different quinolones across various pediatric age groups. This study aims to conduct a comprehensive exploration of ADE in minors using quinolones, utilizing the FDA Adverse Event Reporting System (FAERS) as a foundation, to furnish a safety reference for the clinical administration of these drugs in pediatric patients.

## 2 Materials and methods

### 2.1 Data sources and processing

According to the PUBMED age grouping standards (26), the population was divided into four age groups: infants (0–2 years, not including 2 years), preschool children (2–6 years, not including 6 years), children (6–13 years, not including 13 years), and adolescents (13–18 years, including 18 years). Data from the first quarter of 2015 to the third quarter of 2023, totaling 35 quarters, were extracted. Using MySQL 8.0 for data processing, reports with the generic names or drug components “CIPROFLOXACIN,” “LEVOFLOXACIN,” “MOXIFLOXACIN,” and “OFLOXACIN” identified as the “primary suspect drug” were selected for retrospective pharmacovigilance studies. ADE mentioned in this study were categorized using the preferred system organ class (SOC) and preferred term (PT) from the Medical Dictionary for Regulatory Activities (MedDRA 26.0) (27, 28).

### 2.2 Data analysis

Signal mining for adverse events essentially involves determining whether the reporting frequency of a target adverse event for a specific drug is higher than expected, thereby establishing a statistical link between the drug and the adverse event. The proportional reporting ratio (PRR) and reporting odds ratio (ROR) methods are straightforward, easy to calculate, and understand, but they are prone to generating false positive signals. The ROR method, compared to the PRR method, can reduce the occurrence of false positive signals because it does not require a control group (29–32). PRR is an early method for quantitative analysis of spontaneous reporting systems (SRS) and is the earliest and most basic signal detection algorithm. However, its drawback is that it is difficult to interpret the results when the number of reports is low, which can easily lead to false positive signals. Due to its limitations related to the number of reports, PRR is often used in conjunction with other data mining methods. The Comprehensive Standard Method (MHRA) has adjusted the detection criteria for PRR, considering multiple indicators (PRR, X^2^, and a value) as conditions for signal generation (29).

Based on the four-grid tables of the proportional imbalance method, the reporting odds ratio (ROR) and the integrated standard method (MHRA) were used to calculate the ROR, proportional reporting ratio (PRR), and X^2^ equivalence. To avoid false-positive signals, PT were defined as effective signals only when the calculated values reached the set thresholds (see Table 1) (33–36). Higher ROR and PRR values indicate stronger signals, suggesting a higher likelihood that the target drug is associated with the target adverse event, though this does not necessarily imply a causal relationship (37). All statistical analyses were performed using GraphPad Prism 8(GraphPad Sofware, CA, USA) and Microsoft Excel software. Calculation methods are shown in Tables 1, 2.

**TABLE 1:** Formulas and thresholds of ROR and PRR methods.

Method	Formula	Threshold
ROR Method	$ROR=\frac{a/c}{b/d}$ $95\%CI=e^{\ln\left(R\,O\,R\right)\pm 1.96}\sqrt{(\frac{1}{a}}+\frac{1}{b}+\frac{1}{c}+\frac{1}{d})$	a ≥ 3, Lower bound of 95% CI for ROR > 1 Considered a valid signal
MHRA Method	$PRR\text{ = }\frac{\text{a/}\left(\text{a+b}\right)}{\text{c/}\left(\text{c+d}\right)}$ $X^{2}=\frac{{\left(ad-bc\right)}^{2}\left(a+b+c+d\right)}{\left(a+b\right)\left(c+d\right)\left(a+c\right)\left(b+d\right)}$	a ≥ 3, PRR ≥ 2, X^2^≥ 4Indicative of a valid signal

**TABLE 2:** Fourfold table of disproportional method.

Drug	Number of Target Adverse Event Reports	Number of Other Adverse Event Reports	Total
Target Drug	a	b	a+b
Other Drugs	c	d	c+d
Total	a+c	b+d	N = a+b+c+d

## 3 Results

### 3.1 Basic information on adverse event reports

Among minors, the majority of adverse drug event (ADE) cases reported involved ciprofloxacin (575 cases) and levofloxacin (477 cases). The gender distribution of patients with known sex was relatively balanced, with both male and female patients comprising approximately 40–50% of the cases. ADE reports for ciprofloxacin and moxifloxacin were predominantly from the adolescent group (48.52% and 57.66%, respectively). Ofloxacin ADE reports were similar between the infant group and the preschool children group (30.36% and 32.14%, respectively), while levofloxacin ADE reports were more frequent in the preschool children group (44.30%). The majority of reports were submitted by healthcare professionals (ciprofloxacin 73.04%, moxifloxacin 76.58%, Ofloxacin 57.14%, levofloxacin 85.01%), and most reports originated from the United States. Detailed basic information on the reports is shown in Table 3.

**TABLE 3:** Basic information of ADE reports related to FQs.

Information	Category	Reported Cases [n (%)]			
		Ciprofloxacin	Moxifloxacin	Ofloxacin	Levofloxacin
Cases		575	111	56	447
Gender	Male	237 (41.22%)	52 (46.85%)	28 (50.00%)	187 (41.83%)
	Female	314 (54.61%)	53 (47.75%)	25 (44.64%)	254 (56.82%)
	Unknown	24 (4.17%)	6 (5.41%)	3 (5.36%)	6 (1.34%)
Age Group	Infants	97 (16.87%)	13 (11.71%)	17 (30.36%)	58 (12.98%)
	Preschool Children	65 (11.30%)	14 (12.61%)	18 (32.14%)	198 (44.30%)
	Children	134 (23.30%)	20 (18.02%)	8 (14.29%)	57 (12.75%)
	Adolescents	279 (48.52%)	64 (57.66%)	13 (23.21%)	134 (29.98%)
Reporter	Healthcare Providers	420 (73.04%)	85 (76.58%)	32 (57.14%)	380 (85.01%)
	Non-Healthcare	140 (24.35%)	25 (22.52%)	23 (41.07%)	51 (11.41%)
	Unknown	15 (2.61%)	1 (0.90%)	1 (1.79%)	16 (3.58%)
Top3 Reported Countries		US [192 (33.39%)]	US [28 (25.23%)]	US [28 (50.00%)]	US [204 (45.64%)]
		GB [78 (13.57%)]	FR [8 (7.21%)]	DE [6 (10.71%)]	IT [48 (10.74%)]
		FR [57 (9.91%)]	CA [8 (7.21%)]	FR [5 (8.93%)]	FR [24 (5.37%)]

US, United States; GB, Great Britain; FR, France; CA, Canada; DE, Germany; IT, Italy.

### 3.2 SOC involvement of ADE with different drugs in the same age group

**FIGURE 1:**
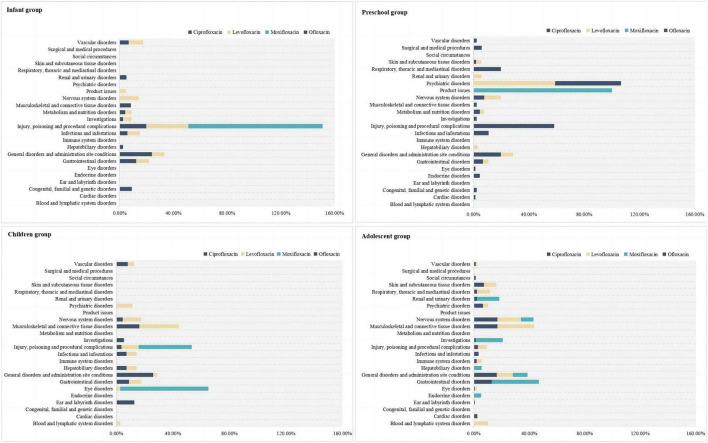
Composition ratio of SOCs with different drugs in patients of the same age group.

In the infant group, ADE from the use of these drugs primarily involved various injuries, poisonings, and procedural complications, with all detected moxifloxacin ADE signals falling within this system (100.00%). In the preschool children group, ciprofloxacin ADE mainly involved respiratory, thoracic and mediastinal disorders (19.72%), as well as general disorders and administration site conditions (19.72%). Levofloxacin primarily affected psychiatric disorders (59.00%), moxifloxacin was mainly associated with product issues (100.00%), and ofloxacin ADE primarily involved various injuries, poisonings, and procedural complications (52.38%) and psychiatric disorders (47.62%). In the children group, ciprofloxacin ADE mainly involved general disorders and administration site conditions (26.13%), levofloxacin primarily affected musculoskeletal and connective tissue disorders (28.07%), and moxifloxacin ADE involved eye disorders (62.50%). In the adolescent group, ciprofloxacin ADE primarily involved nervous system disorders (17.11%), musculoskeletal and connective tissue disorders (17.11%), and general disorders and administration site conditions (16.63%). Levofloxacin primarily affected musculoskeletal and connective tissue disorders (26.50%), and moxifloxacin mainly involved gastrointestinal disorders (33.93%). For more details, see Figure 1.

### 3.3 Distribution of PT in the main SOC affected by different drugs in each age group

From the use of different drugs in the same age group, it can be observed that quinolones antibiotics exhibit distinct characteristics under various SOC such as injuries, poisonings, and procedural complications, psychiatric disorders, eye disorders, and gastrointestinal disorders. The following sections will further analyze the PT signals for these SOC.

#### 3.3.1 Distribution of PT for injuries, poisonings, and procedural complications

**FIGURE 2:**
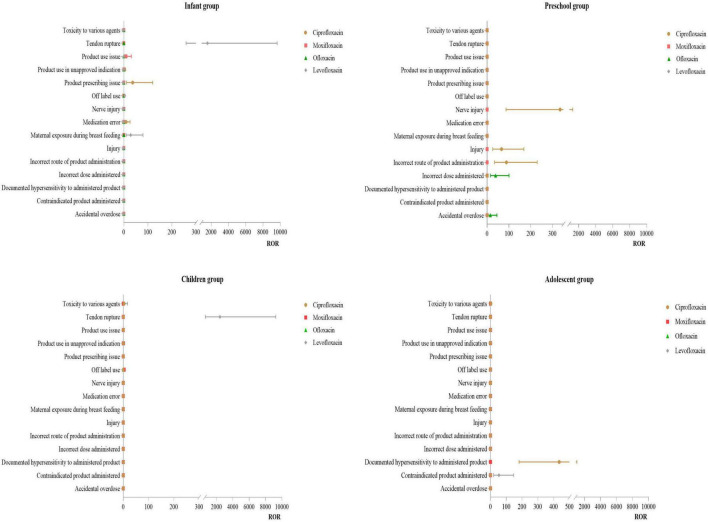
PT distribution of various injuries, poisoning and operation complications.

Infant Group: For ciprofloxacin, the primary PT was product prescription issues (ROR:36.92). For levofloxacin, the main of PT were tendon rupture (ROR:1579.91) and exposure during breast feeding (ROR:28.53). Preschool Group: For ciprofloxacin, the primary PT were nerve injury (ROR:334.82) and incorrect route of drug administration (ROR:88.64). Children Group: For levofloxacin, the main PT was tendon rupture (ROR:2417.07). Adolescent Group: For ciprofloxacin, the primary PT was documented hypersensitivity to the administered product (ROR:435.41). For more details, see Figure 2.

#### 3.3.2 Distribution of PT for psychiatric disorders

**FIGURE 3:**
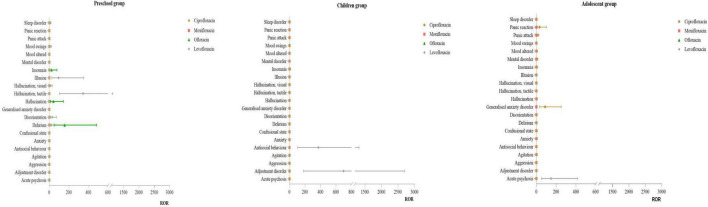
PT distribution of psychiatric disorders.

Infant Group: No effective signals were detected for this SOC. Preschool Group: For levofloxacin, the primary PT were hallucination, tactile (ROR: 345.17) and illusion (ROR:94.40). For ofloxacin, the main PT were delirium (ROR:156.47) and hallucination (ROR:41.78). Children Group: For levofloxacin, the primary PT were adjustment disorder (ROR:698.73) and antisocial behavior (ROR:372.62). No effective signals were detected for other drugs. Adolescent Group: For ciprofloxacin, the main PT was generalized anxiety disorder (ROR:88.33). For levofloxacin, the primary PT was acute psychosis (ROR:147.84). For more details, see Figure 3.

#### 3.3.3 Distribution of PT for eye disorders

**FIGURE 4:**
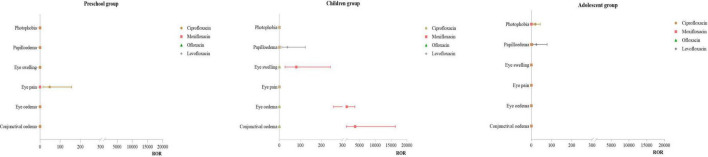
PT distribution of eye organ diseases.

Infant Group: No effective signals were detected for this SOC. Preschool Group: For ciprofloxacin, the primary PT was eye pain (ROR:47.79). Children Group: For moxifloxacin, the primary PT were conjunctival edema (ROR:3618.39), eye edema (ROR:952.08), and eye swelling(ROR:80.61). For levofloxacin, the main PT was papilloedema (ROR:38.23). Adolescent Group: For levofloxacin, the primary PT was papilloedema (ROR:20.50). For more details, see Figure 4.

#### 3.3.4 Distribution of PT for gastrointestinal disorders

**FIGURE 5:**
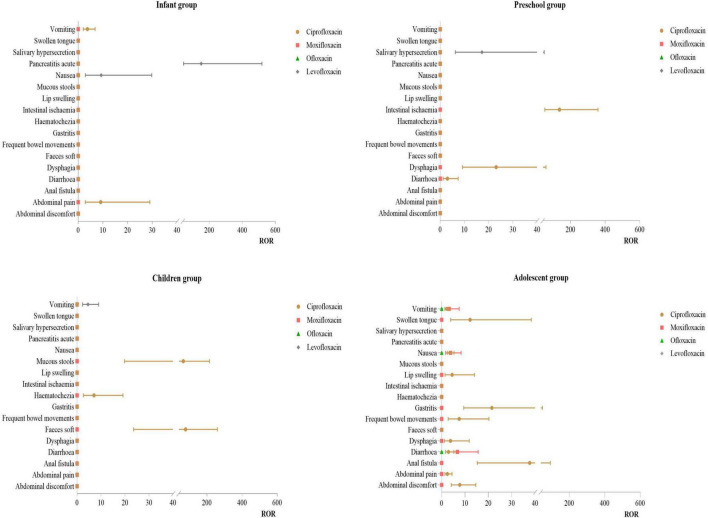
PT distribution of gastrointestinal diseases.

Infant Group: For ciprofloxacin, the primary PT was abdominal pain (ROR:9.11). For levofloxacin, the main PT were pancreatitis acute (ROR:150.42) and nausea (ROR:9.27). Preschool Group: For ciprofloxacin, the primary PT were intestinal ischemia (ROR: 135.61) and dysphagia (ROR: 23.10). For levofloxacin, the main PT was salivary hypersecretion (ROR:17.23). Children Group: For ciprofloxacin, the primary PT were faces soft (ROR:78.15) and mucous stools (ROR:65.12). Adolescent Group: Ciprofloxacin had multiple effective signals, including anal fistula (ROR:37.67), gastritis (ROR:21.49), and swollen tongue (ROR:12.19). For moxifloxacin, the primary PT was diarrhea (ROR:6.75). For more details, see Figure 5.

## 4 Discussion

This study found that the number of adverse reactions reported for ciprofloxacin and moxifloxacin was higher in the adolescent group, while the number of adverse reactions reported for ofloxacin and levofloxacin was higher in the preschool children group. This discrepancy may be related to the approved indications for these drugs. Currently, the FDA has approved pediatric indications for these quinolones: ciprofloxacin can be used for children from birth to 17 years old for post-exposure inhalational anthrax and plague, as well as for severe/complicated urinary tract infections or complicated pyelonephritis in children aged 1 to 17 years (7); levofloxacin can be used for children over 6 months old for post-exposure inhalational anthrax and plague (8). The majority of reports were submitted by healthcare professionals, indicating that clinical healthcare providers are highly concerned about the safety of quinolones use in minor patients. The geographical distribution of the reports was predominantly from the United States, likely due to the data source used in this study.

Patients in different age groups exhibited distinct safety profiles when using quinolones antibiotics. This not only reveals potential age-related drug reaction patterns but also underscores the importance of individualized drug selection in clinical practice. Tailoring antibiotic therapy to the specific needs and risks of different pediatric age groups is crucial for optimizing treatment efficacy and minimizing adverse effects. The findings of this study emphasize the need for heightened clinical awareness and vigilance when prescribing quinolones to younger populations, ensuring that therapeutic decisions are informed by both the benefits and potential risks associated with these medications.

### 4.1 Injuries, poisonings, and procedural complications

In the infant group, the use of ciprofloxacin was primarily associated with prescription issues and medication errors. This may be related to the pharmacokinetics of ciprofloxacin differing between infants and other age groups. Patel K studies (38) have shown that the half-life of ciprofloxacin in infants under 1 year is longer than in children aged 1–5 years. Younger children have lower oral bioavailability of ciprofloxacin compared to older children and adults; thus, the recommended dosing frequency is often q12h or q8h. If clinicians overlook this characteristic, it may lead to more prescription issues or medication errors. Our study found that cases of tendon rupture mainly occurred with moxifloxacin in the infant group and with levofloxacin in the children group. This might be related to age group differences. Studies (39, 40) have indicated that the risk of tendon rupture and tendinitis in adolescent patients using quinolones is very low. Most data do not show significant musculoskeletal sequelae associated with quinolones use in neonates, infants, and children. Musculoskeletal adverse events observed in children are usually joint pain, which typically resolves after discontinuation or treatment without significant sequelae. However, our study suggests that there is a need to be vigilant about these risks in the infant and children groups. Given that infants have limited ability to express their discomfort, they are at risk of accumulating injuries, leading to severe adverse events. Therefore, when treating infants with quinolones antibiotics, clinicians should fully consider the physiological characteristics of the drug and the physiological functions of the patient, selecting appropriate dosages, dosing frequencies, and durations of therapy.

### 4.2 Psychiatric disorders

No effective ADE signals for psychiatric disorders were detected in the infant group, which could be due to the limited use of these drugs in this age group or the infants’ restricted ability to express and exhibit symptoms. In the preschool group, levofloxacin-related psychiatric adverse events primarily included tactile hallucinations and illusions, while ofloxacin-related events mainly included delirium and hallucinations. In other age groups, psychiatric disorders associated with levofloxacin included adjustment disorders, antisocial behavior, and acute psychosis. Our study suggests that the use of levofloxacin in minors requires attention to the potential for psychiatric disorders. Literature and research (20, 33) indicate that the substitution at the C7 position of quinolones is related to their blocking effect on gamma-aminobutyric acid (GABA) receptors. Levofloxacin, which has a methylpiperazine ring at the C7 position, increases the drug’s lipophilicity and tissue penetration. This results in higher concentrations in the cerebrospinal fluid, where it can inhibit the binding of γ-GABA to its receptors in brain tissue, leading to increased central nervous system excitability and subsequent psychiatric symptoms (41).

Moreover, the blood-brain barrier (BBB) is largely developed by around three years of age. Before the BBB is fully developed, these drugs can more easily penetrate brain tissue, causing psychiatric symptoms. Clinicians should closely monitor the mental state of preschool children (≤ 3 years old) when using these drugs, promptly assess any discomfort, and advise caregivers to observe the children’s mental state closely.

### 4.3 Eye disorders

The ADE in this system were primarily concentrated in the children group using moxifloxacin, mainly manifesting as conjunctival edema and eye swelling. These findings are consistent with results from related studies (42–47). The mechanism of moxifloxacin-induced ocular toxicity may be related to its high tissue affinity and uptake rate, particularly in pigmented tissues such as the iris (48). Moxifloxacin can form complexes with melanin, potentially leading to drug accumulation in melanin-rich tissues (such as the iris), thereby causing toxic effects (49). Additionally, based on data showing increased photosensitivity of the skin following moxifloxacin administration, it is hypothesized that the drug has a direct toxic effect on iris pigment, which can cause the release of iris pigment and subsequently trigger inflammatory responses (50). This finding suggests that clinicians should closely monitor the ocular health of pediatric patients when using moxifloxacin and take prompt action if signs of adverse reactions appear.

### 4.4 Gastrointestinal disorders

Our study found that gastrointestinal system ADE in minors using quinolones were primarily associated with ciprofloxacin and mainly concentrated in the adolescent group, presenting as anal fistula, gastritis, and swollen tongue. This may be related to the higher usage of ciprofloxacin in this age group. The primary connective tissue in the gastrointestinal tract is collagen (51, 52), and fluoroquinolones can reduce collagen synthesis by increasing the expression of matrix metalloproteinases, which can degrade collagen (53–55). Additionally, fluoroquinolones have chelating properties for metal ions (such as calcium, magnesium, and aluminum) and show higher affinity for ions in connective tissue than in serum (56–58), potentially promoting collagen degradation by chelating these metal ions. Animal studies (59) have shown that fluoroquinolones can specifically antagonize GABAA receptors on the vagus nerve, indicating that the vagus nerve might be particularly susceptible to the toxicity of these drugs. Changes in the microbiome can affect the gut-brain axis via the vagus nerve and induce functional gastrointestinal disorders (60, 61). Thus, the possibility that fluoroquinolones induce functional gastrointestinal diseases by directly damaging the vagus nerve and disrupting the host microbiome should not be overlooked (62). All quinolones are known to cause mild gastrointestinal reactions, a common adverse effect of this drug class. However, in our study, gastrointestinal system ADE were primarily observed in the adolescent group using ciprofloxacin. This may be related to the FDA-approved pediatric indications for quinolones, which only approve ciprofloxacin for use in children aged 1–17 years for severe/complicated urinary tract infections and complicated pyelonephritis. Therefore, when using these drugs in adolescent patients, gastrointestinal function should be closely monitored, and thorough medication education should be provided to reduce the occurrence of adverse reactions.

### 4.5 Study limitations

This study utilized the extensive data from FAERS to overcome the limitations of clinical trials, such as limited sample sizes and short observation periods, aiming to obtain research results reflective of real-world conditions. However, this method also has its limitations. Given that the FAERS database is a spontaneous, retrospective reporting system, there may be selection and recall biases, potentially leading to the underestimation or overestimation of certain adverse reactions. Additionally, the FAERS database lacks detailed information on comorbidities and other potential health impact factors, which limits our ability to control for confounding factors in data analysis (35–38). Although the ADE signals detected in this study statistically suggest an association between fluoroquinolone drugs and the ADEs, this does not suffice to prove a biological causal relationship, which requires further verification through in-depth clinical trials (63). Lastly, since the FAERS database does not provide the specific total number of drug users, we can currently only estimate the strength of the association between the drug and adverse events, and cannot clearly link the high incidence of ADEs to the high usage rate of the drug in specific age groups (63).

## 5 Conclusion

The occurrence of adverse events in minors using quinolones antibiotics varies across different age groups. In clinical practice, it is essential to strictly adhere to the indications for quinolones use, selecting appropriate dosages, frequencies, and durations of administration while avoiding long-term use. Close attention should be paid to potential short-term adverse reactions, such as various injuries, poisonings, psychiatric disorders, eye disorders, and gastrointestinal adverse reactions. Long-term follow-up may be necessary in some cases. Additionally, it is crucial to strengthen pharmaceutical services, including medication education and monitoring, to reduce the incidence of ADE.

## Data Availability

The raw data supporting the conclusions of this article will be made available by the authors, without undue reservation.
